# M2 Polarization of Human Macrophages Favors Survival of the Intracellular Pathogen *Chlamydia pneumoniae*

**DOI:** 10.1371/journal.pone.0143593

**Published:** 2015-11-25

**Authors:** Tanja Buchacher, Anna Ohradanova-Repic, Hannes Stockinger, Michael B. Fischer, Viktoria Weber

**Affiliations:** 1 Christian Doppler Laboratory for Innovative Therapy Approaches in Sepsis, Danube University Krems, Krems, Austria; 2 Molecular Immunology Unit, Institute for Hygiene and Applied Immunology, Center for Pathophysiology, Infectiology and Immunology, Medical University of Vienna, Vienna, Austria; 3 Department of Blood Group Serology and Transfusion Medicine, Medical University of Vienna, Vienna, Austria; 4 Department for Health Sciences and Biomedicine, Danube University Krems, Krems, Austria; Midwestern University, UNITED STATES

## Abstract

Intracellular pathogens have developed various strategies to escape immunity to enable their survival in host cells, and many bacterial pathogens preferentially reside inside macrophages, using diverse mechanisms to penetrate their defenses and to exploit their high degree of metabolic diversity and plasticity. Here, we characterized the interactions of the intracellular pathogen *Chlamydia pneumoniae* with polarized human macrophages. Primary human monocytes were pre-differentiated with granulocyte macrophage colony-stimulating factor or macrophage colony-stimulating factor for 7 days to yield M1-like and M2-like macrophages, which were further treated with interferon-γ and lipopolysaccharide or with interleukin-4 for 48 h to obtain fully polarized M1 and M2 macrophages. M1 and M2 cells exhibited distinct morphology with round or spindle-shaped appearance for M1 and M2, respectively, distinct surface marker profiles, as well as different cytokine and chemokine secretion. Macrophage polarization did not influence uptake of *C*. *pneumoniae*, since comparable copy numbers of chlamydial DNA were detected in M1 and M2 at 6 h post infection, but an increase in chlamydial DNA over time indicating proliferation was only observed in M2. Accordingly, 72±5% of M2 *vs*. 48±7% of M1 stained positive for chlamydial lipopolysaccharide, with large perinuclear inclusions in M2 and less clearly bordered inclusions for M1. Viable *C*. *pneumoniae* was present in lysates from M2, but not from M1 macrophages. The ability of M1 to restrict chlamydial replication was not observed in M1-like macrophages, since chlamydial load showed an equal increase over time for M1-like and M2-like macrophages. Our findings support the importance of macrophage polarization for the control of intracellular infection, and show that M2 are the preferred survival niche for *C*. *pneumoniae*. M1 did not allow for chlamydial proliferation, but failed to completely eliminate chlamydial infection, giving further evidence for the ability of *C*. *pneumoniae* to evade cellular defense and to persist in human macrophages.

## Introduction

Macrophages, the first line of host defense against invading microbes, are frequently targeted by intracellular pathogens that escape eradication by internalization, using antigen-presenting cells as niche for survival and replication. It may seem paradoxical that macrophages, the very cells equipped for pathogen destruction, provide a favorable environment for the lifecycle of a number of bacteria. An intracellular existence in macrophages, however, may be appealing for various reasons, e.g. to avoid pathogen recognition by antibodies and attack by aggressive effector molecules, or to exploit the high degree of metabolic diversity and plasticity of macrophages [1]. Pathogens have evolved a number of different strategies to survive in macrophages [2, 3], and cross-talk between intracellular pathogens and their host cells occurs within various membrane-bound compartments or within the cytosol, depending on the nature of the invading pathogen.

*Chlamydia pneumoniae* is a Gram-negative, obligate intracellular bacterium which causes respiratory infections, such as acute pneumonia [4] and has been associated with a number of chronic diseases including atherosclerosis or Alzheimer’s disease [5–7]. *Chlamydiae* exhibit a unique dimorphic developmental cycle with extracellular infectious elementary bodies and intracellular non-infectious reticulate bodies. In response to external triggers, *C*. *pneumoniae* may enter a state of persistence, allowing the pathogen to ride out hostile conditions while maintaining a long-term, chronic infection within membrane-bound compartments in the cytoplasm of its host cells [4, 8]. There is evidence for the persistence of *C*. *pneumoniae* in primary human monocytes [9] and for their ability to replicate in monocyte-derived macrophages [10, 11], but the interdependence of chlamydial infection and macrophage polarization is unknown.

Activated macrophages are broadly classified into pro-inflammatory (M1) and anti-inflammatory (M2) macrophages. The M1 phenotype is commonly induced by bacterial components such as LPS and by the T_H_1 cytokine interferon-γ (IFN-γ), resulting in a pro-inflammatory response with microbicidal and tumoricidal capacity, while M2 are induced in response to the T_H_2 cytokines IL-4 and /or IL-13 and mainly participate in parasite clearance, damping of inflammation and tumor progression, tissue remodeling, and immunoregulation [12–14]. Existing literature supports the idea that M2 macrophages might provide hospitable niches for pathogen survival and replication due to their reduced antimicrobial capacity and nutrient rich environment [15, 16]. Consequently, some species, such as *Mycobacterium tuberculosis*, *Listeria monocytogenes*, and *Francisella tularensis* have been shown to induce M2 phenotype characteristics in host macrophages to suit their own requirements [17–21]. On the other hand, it is established that intracellular pathogens have evolved strategies to interfere with M1 polarization and to neutralize M1-related effectors [22]. A recent study in a murine model has shown that the intracellular survival and persistence of *C*. *muridarum* in mouse macrophages is determined by their phenotypic plasticity [23], but it remains open whether this holds also true for human *Chlamydiae* infecting polarized macrophages. Here, we investigated the ability of *C*. *pneumoniae* to infect and survive in polarized human macrophages and demonstrate that polarization towards the M2 phenotype favors survival and replication of *C*. *pneumoniae*.

## Materials and Methods

### Ethics statement

Studies with human blood monocytes were approved by the ethics committee of the Medical University Vienna (ECS2177/2013). Monocytes were obtained from healthy individuals eligible for single donor platelet apheresis in a blood bank setting. Written informed consent was obtained from all donors prior to the onset of the study.

### Cell culture media and reagents

Phosphate-buffered saline (PBS), gentamicin, amphotericin B, macrophage serum free medium (MSF) were obtained from Invitrogen (Lofer, Austria). RPMI-1640, human male AB serum (sterile-filtered), 4-(2-hydroxyethyl)-1-piperazineethanesulfonic acid (HEPES), fetal bovine serum (FBS), accutase, interleukin-4 (IL-4), interferon-γ (IFN-γ), and lipopolysaccharide (LPS) from *E*. *coli* (055:B5, purified by gel filtration) were purchased from Sigma-Aldrich (St Louis, MO, USA). Granulocyte macrophage colony-stimulating factor (GM-CSF) was purchased from Novartis (Basel, Switzerland) and macrophage colony-stimulating factor (M-CSF) from Peprotech, Rocky Hill, NJ.

### Propagation of C. pneumoniae

*C*. *pneumoniae* strain CWL-029 was obtained from the American Type Culture Collection (ATCC, VR-1310) and propagated in the human epithelial cell line HEp-2 (ATCC, CCL23) as previously reported [9]. For chlamydial quantification, serially diluted elementary bodies (EBs) were inoculated onto uninfected HEp-2 cells in 8-well μ-slides (ibidi, Martinsried, Germany) which were cultured the day before, incubated for 48 h at 35°C, 5% CO_2_, and fixed in cold methanol for 10 min, followed by immunofluorescence staining (see below). In order to exclude mycoplasma contamination, cell culture and chlamydial stocks were regularly tested using the Venor^™^GeM *Mycoplasma* Detection Kit, targeting 16S rRNA genes (Minerva, Biolabs, Berlin, Germany).

### Isolation of monocytes

Human peripheral blood mononuclear cells (PBMCs) were isolated from leukocyte reduction system (LRS) chambers of a Trima Accel^®^ automated blood collection system (Terumo BCT, Lakewood, CA) by Ficoll gradient centrifugation, as previously described [9]. Human monocytes were purified from PBMCs by negative selection using the monocyte isolation kit II (Miltenyi Biotec, Bergisch Gladbach, Germany) according to the manufacturer’s instructions. The purity of CD14-positive monocytes was >87% as assessed by flow cytometry.

### Macrophage differentiation

**Fig 1:**
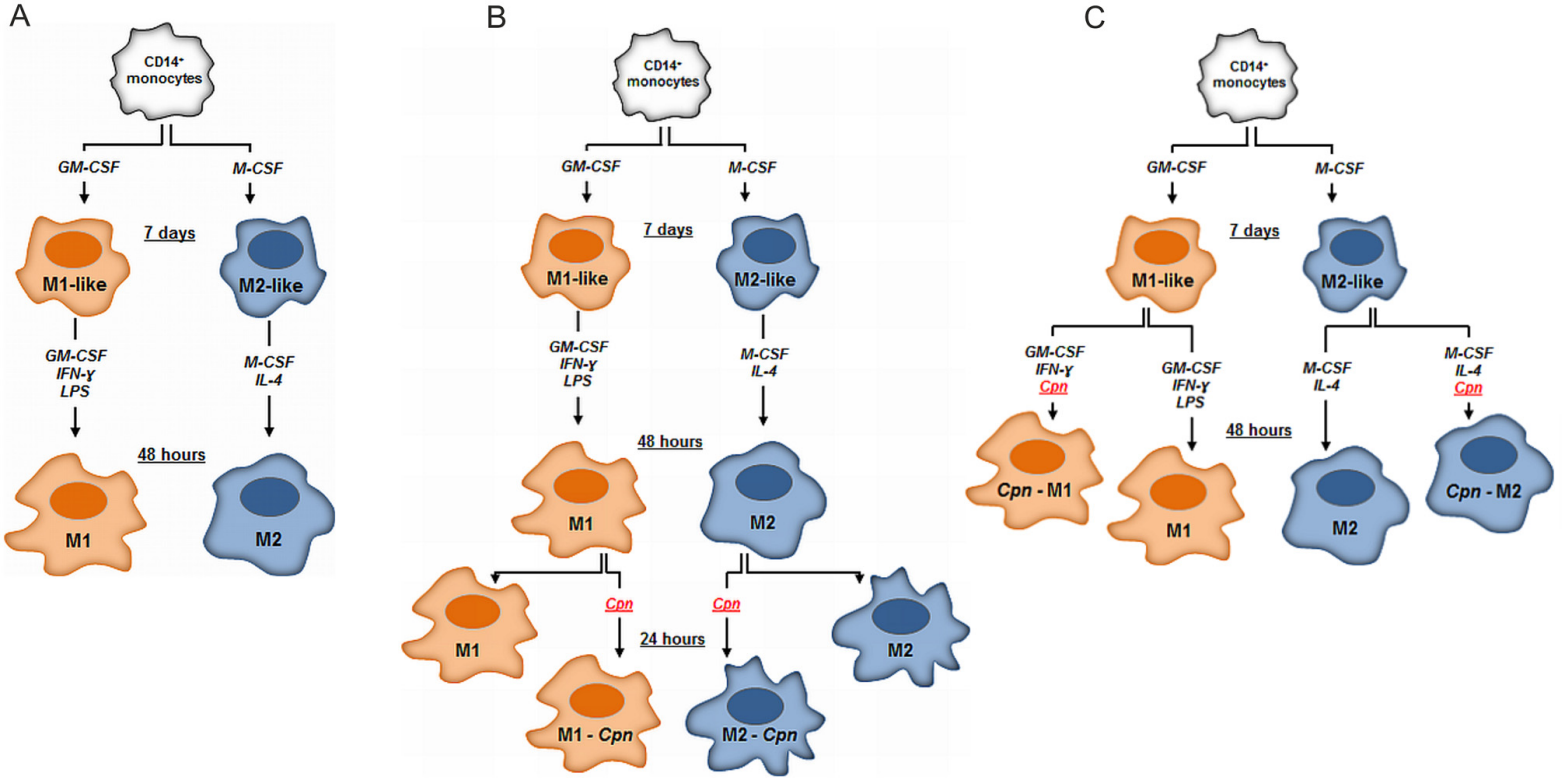
Macrophage Polarization. **(A)** CD14 positive human blood monocytes were treated either with 25 ng/mL GM-CSF or 50 ng/mL M-CSF for 7 days to yield M1-like or M2-like macrophages. M1-like macrophages were activated with GM-CSF, LPS and 50 ng/mL IFN-γ for an additional 48 h to yield M1 macrophages, while M2-like macrophages were treated with M-CSF and IL-4 to yield M2. **(B)** To examine the impact of macrophage polarization on survival and proliferation of *C*. *pneumoniae*, M1 and M2 macrophages were infected and cultured with *C*. *pneumoniae* for 24 h. **(C)** M1-like macrophages were infected with *C*. *pneumoniae* and cultured for 48 h in the presence of GM-CSF and IFN-γ, while infected M2-like macrophages were cultured with M-CSF and IL-4. Uninfected macrophages served as control.

CD14^+^ monocytes were cultured in 6-well plates at a concentration of 1x10^6^ cells/mL in RPMI-1640 medium supplemented with 10% FBS, 20 mM HEPES, 10 μg/mL gentamicin, 0.25 μg/mL amphotericin B, 25 ng/mL GM-CSF or 50 ng/mL M-CSF, respectively, at 37°C in a humidified atmosphere (5% CO_2_). After 7 days, macrophages were detached with accutase and washed with PBS (300 g, 5 min, 4°C). GM-CSF or M-CSF treated macrophages (further designated as M1-like or M2-like) were resuspended at a concentration of 4x10^5^/mL in MSF medium supplemented with 20 mM HEPES and 2% AB serum, under antibiotic free conditions. M1-like macrophages were differentiated into M1 macrophages in the presence of 25 ng/mL GM-CSF, 50 ng/mL IFN-γ and 100 ng/mL LPS for 48 h. M2 macrophages were obtained by culturing M2-like macrophages in the presence of 50 ng/mL M-CSF and 20 ng/mL IL-4 for 48 h (Fig 1A). M0 macrophages were generated from monocytes as described for M1 and M2 macrophages, but without the addition of growth factors or cytokines. Culture supernatants were collected at the respective times, and centrifuged at 600 g for 5 min at 4°C and stored at -80°C for cytokine determination. The percentage of viable M1 and M2 macrophages was >96%, as assessed by gating according to their forward and side scatter characteristics and Annexin-V-negative/PI-negative staining using flow cytometry.

### Infection of polarized macrophages with *C*. *pneumoniae*

For infection of polarized macrophages, 4x10^4^ chlamydial inclusion forming units (IFU) per well were added to 4x10^5^ M1 and M2 macrophages and co-cultured in a final volume of 1 mL medium in 12 well plates at 37°C in 5% CO_2_ (Fig 1B). Mock controls were prepared following the propagation, harvest and purification procedure for EBs [24, 25], but in the absence of chlamydial infection. Culture supernatants were collected 24 h after infection, centrifuged immediately thereafter, and stored at −80°C until quantification of cytokines. Cells were processed for real-time quantitative PCR to quantify chlamydial load (see below).

To investigate the capacity of *C*. *pneumoniae* to infect M1-like and M2-like macrophages and to induce further macrophage polarization, M1-like and M2-like macrophages were cultured in MSF-medium supplemented with 2% AB serum in the presence of either GM-CSF and IFN-γ or M-CSF and IL-4 with and without addition of *C*. *pneumoniae* (4x10^4^ IFU/well in a final volume of 1 mL in 12 well plates) at 37°C in 5% CO_2_ (Fig 1C). Culture supernatants were collected after 48 h, centrifuged and stored at −80°C until quantification of cytokines. Cells were processed for real-time PCR to quantify chlamydial load.

### Recovery assay

Fully polarized macrophages (8x10^5^) exposed to *C*. *pneumoniae* (8x10^4^ IFU) were washed with PBS, and cells were scraped and vortexed with zirconium dioxide beads. Chlamydial bodies were obtained from the lysates by centrifugation as described [9] and passaged once onto 8x10^4^ HEp-2 cells. At 48 hours post infection, HEp-2 cells were fixed and stained for immunofluorescence [9].

### Real-time quantitative PCR

Isolation and quantification of DNA was performed as described [9]. In brief, *C*. *pneumoniae* genomes were quantified by real-time PCR, targeting a 222 bp sequence on *Chlamydia* 16S rRNA. The oligonucleotide primers and TaqMan probes were synthesized by Microsynth AG (Balgach, Switzerland) and used as described in detail previously [26]. Real-time PCR reactions were performed in a final volume of 20 μL including 2.5x Master Mix and Taq polymerase (Mastermix 16S, Molzym, Bremen, Germany), forward primer (0.75 μM), reverse primer (0.75 μM), FAM-TAMRA probe (0.75 μM) and 2 ng of DNA. Amplification and detection were performed for 10 min at 95°C, followed by 50 cycles of 10 s at 95°C and 65 s at 60°C. Standards of known concentration (10^1^, 10^2^, 10^3^, 10^4^, 10^5^ and 10^6^ copies) were prepared for the 16S rDNA target gene from PCR amplified *C*. *pneumoniae* DNA of infected macrophages by conventional PCR, and purified with a QIAmp Mini DNA kit according to the manufacturer’s instructions. Samples were run in triplicate and all reactions were carried out using the iCycler IQ system (BioRad, Vienna, Austria).

### Immunofluorescence

To visualize chlamydial inclusions, cells were directly stained with FITC-conjugated anti-*Chlamydia*-LPS monoclonal antibody and human cells were counterstained with Evans Blue (Pathfinder, Bio-Rad, Vienna, Austria) according to the manufacturer’s protocol. Nuclei were stained with 1.5 μM 4',6-diamidino-2-phenylindole (DAPI, Sigma Aldrich, MO, USA) and fluorescence images were acquired with a Zeiss LSM 700 laser scanning confocal microscope (Carl Zeiss, Jena, Germany) using a 40 x oil objective (numerical aperture 1.3) or a 63 x oil objective (numerical aperture 1.4). Percentages of *C*. *pneumoniae* positive cells were calculated by counting a minimum of 100 cells per slide.

### Flow cytometry

The purity of isolated monocytes was examined by determination of CD14 positive cells as described [9]. Macrophage subsets were detached with ice cold PBS containing 1.5 mM EDTA and washed with PBS. 5x10^5^ macrophages per 50 μL PBS/2%FBS were stained with 5 μL PE-conjugated CD14 (clone RMO52), CD11b (clone D12), CD86 (clone 2331), HLA-DR (clone L243), CD163 (clone GHI/61), CD209 (clone 9E9A8) and FITC-conjugated CD206 (clone 19.2) or with the appropriate isotype control antibodies (BD Biosciences). After one washing step, marker expression was analyzed on an FC 500 flow cytometer (Beckman Coulter), and data were analyzed using the FlowJo software (Tree Star Inc, Ashland, OR). Living macrophages were gated according to their forward- and side scatter characteristics and apoptotic or dead cells were excluded using the Annexin V-FITC/PI apoptosis detection kit (BD Biosciences). Data are shown as mean fluorescence intensity (MFI) from three independent experiments.

### Quantification of cytokines

Tumor necrosis factor-α (TNF-α), interleukin (IL)-1β, IL-6, IL-12p70, IL-12p40, IL-10, CCL17 and CCL24 were determined in culture supernatants using the Bio-Plex 200 system (Bio-Rad, Vienna, Austria).

### Statistical analysis

Statistical analysis was performed using the software package SPSS Statistics for Windows, version 18.0 (SPSS Inc., Chicago, Illinois, USA). When comparing two groups, data were analyzed by the nonparametric Wilcoxon rank sum test. Data are expressed as means ± SD. Significance was accepted at P ≤ 0.05.

## Results

### M1 and M2 macrophages differ with respect to morphology, surface marker expression, as well as cytokine and chemokine secretion

**Fig 2:**
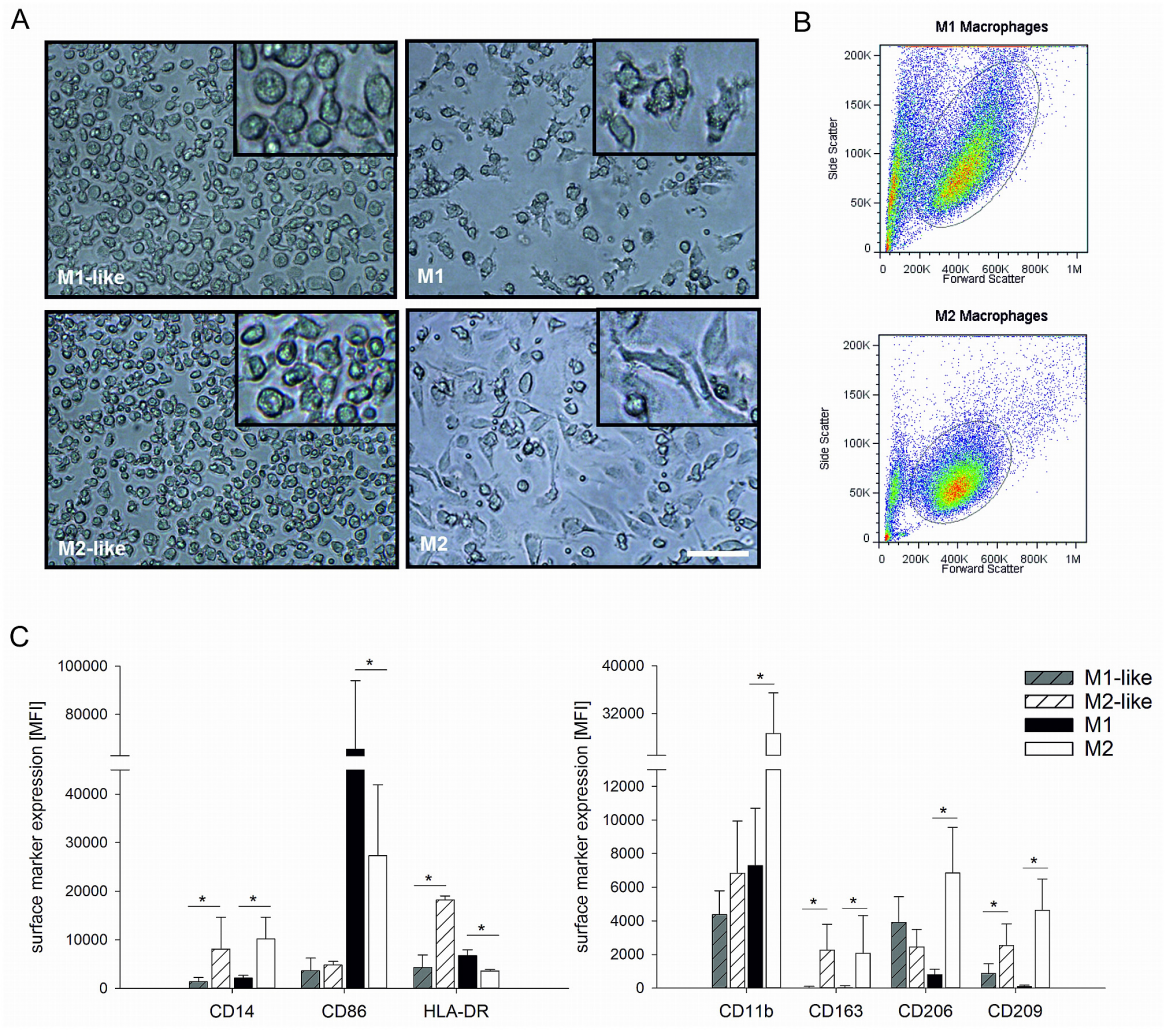
Morphology and surface marker expression of polarized macrophages. Monocytes were cultured and polarized as shown in Scheme 1A to generate M1-like, M2-like, M1, and M2 macrophages. Morphology was assessed by light microscopy (panel A, scale bar 100 μm) and by flow cytometry according to forward/side scatter characteristics (panel B). The expression of surface markers was determined by flow cytometry (panel C). Data are expressed as mean ± SD for 3 independent experiments. MFI, mean fluorescence intensity.

M1-like and M2-like macrophages (obtained by treatment of monocytes with GM-CSF or M-CSF, respectively, for 7 days) exhibited round shape. M1 macrophages (obtained by treatment of M1-like macrophages with LPS and IFN-γ for 48 h) predominantly retained this morphology, whereas the majority of M2 macrophages (obtained by treatment of M2-like macrophages with IL-4) had a spindle-shaped appearance (Fig 2A). M1 macrophages displayed increased size and granularity as compared to M2 cells, as shown by their forward and side scatter characteristics in flow cytometry (Fig 2B).

The different polarization states were associated with distinct surface marker profiles, as revealed by flow cytometric analysis of CD14, CD11b, CD206, CD163, CD209, CD86, as well as HLA-DR surface expression (Fig 2C). The monocyte marker CD14 was retained on M2, but not on M1 macrophages. CD11b, CD163, CD206, and CD209 were highly expressed on M2 macrophages, but to a significantly lesser extent on M1 macrophages. Of note, M1-like macrophages exhibited high CD206 expression, but strong down-regulation occurred on fully polarized M1 macrophages. M1 macrophages displayed significantly higher CD86 and HLA-DR levels as compared to M2. HLA-DR was highly expressed on M2-like macrophages, but was significantly down-regulated on fully polarized M2.

**Fig 3:**
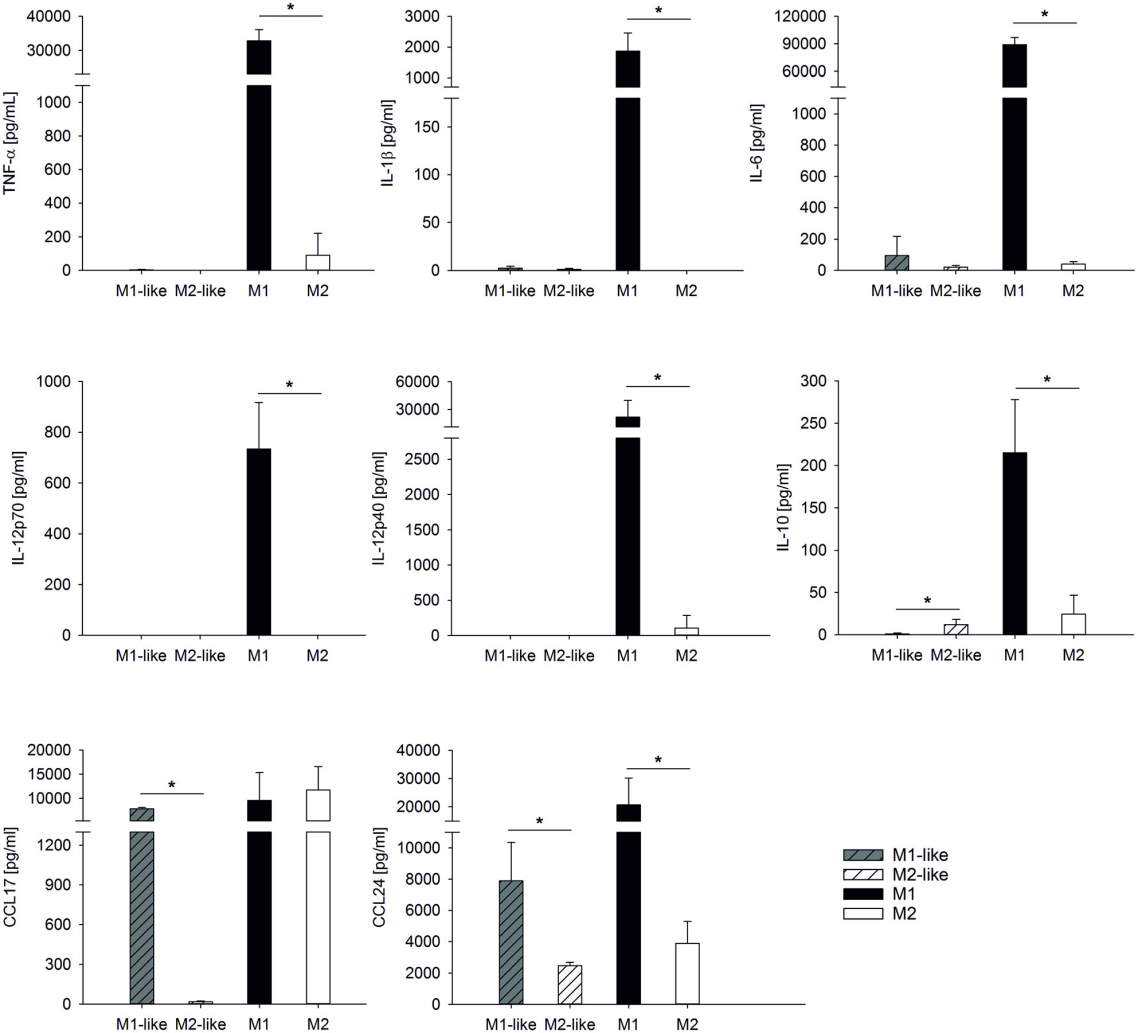
Cytokine secretion of polarized macrophages. Monocytes were cultured and polarized as shown in Scheme 1A to generate M1-like, M2-like, M1, and M2 macrophages. Cytokines were quantified in the culture supernatants of M1-like and M2-like macrophages after 7 days, and in the supernatants of M1 and M2 macrophages after an additional 48 h. Concentrations are expressed as mean ± SD for 3 independent experiments.

M1-like and M2-like macrophages secreted only negligable amounts of TNF-α, IL-1β, IL12p70, and IL-12p40, and very low levels of IL-6 as well as IL-10. M1-like macrophages, however, produced high amounts of the chemokines CCL17 and CCL24. The latter was also secreted by M2-like macrophages, albeit to a lesser extent as compared to M1-like macrophages (Fig 3). Polarization of M1-like and M2-like into M1 and M2 macrophages caused a dramatic change in the array of secreted soluble signals. M1 macrophages released high amounts of TNF-α, IL-1β, IL-6, IL12p70, and IL-12p40, while the secretion of these cytokines was very low or undetectable in M2 macrophages. The high CCL17 secretion observed in M1-like macrophages was not further enhanced by polarization to M1, whereas polarization of M2-like macrophages to M2 was associated with strong upregulation of CCL17.

### *C*. *pneumoniae* favor M2 macrophages as survival niche

**Fig 4:**
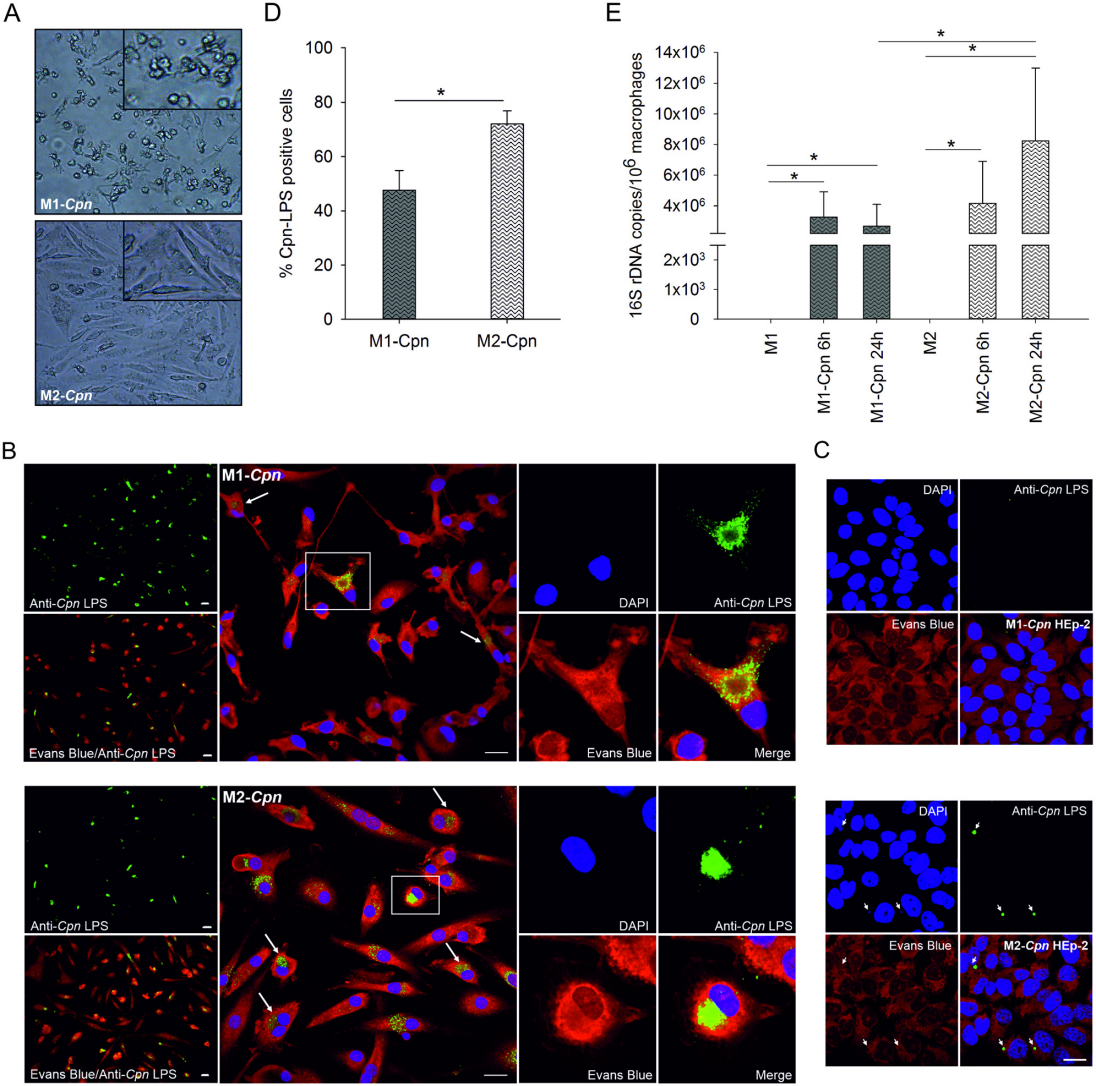
Visualization and quantification of *C*. *pneumoniae* in M1 and M2 macrophages. Monocytes were cultured and polarized as shown in Scheme 1B and fully polarized M1 and M2 macrophages (4x10^5^/mL each) were infected with *C*. *pneumoniae* (4x10^4^ IFU) for 24 h. The morphology of infected M1 and M2 macrophages was determined by light microscopy (panel A, scale bar 100 μm). *C*. *pneumoniae* (green) was detected in M1 and M2 macrophages at 24 h post infection using immunofluorescence (panel B; scale bar = 20 μm). Cells were counterstained with Evans Blue (red), and DNA was visualized with DAPI (blue). Recovery of *C*. *pneumoniae* was evaluated by recultivating disrupted M1 and M2 macrophages 24 h post infection in HEp-2 cells (panel C; scale bar = 20 μm). The percentage of M1 and M2 macrophages that stained positive for *C*. *pneumoniae* at 24 h post infection was calculated by counting a minimum of 100 cells per slide (panel D). *C*. *pneumoniae* 16S rDNA was quantified by real-time PCR in infected M1 and M2 macrophages and in uninfected cells at 6 and 24 h post infection (panel E). Data are expressed as mean ± SD for 3 independent experiments.

To examine the impact of macrophage polarization on the survival and proliferation of the intracellular pathogen *C*. *pneumoniae*, M1 and M2 macrophages were infected and cultured with *C*. *pneumoniae* for 24 h. While infected M1 macrophages retained their round shape, infected M2 drastically changed their morphology into elongated, fibroblast-like, spindle-shaped cells (Fig 4A). M2 contained typical large chlamydial inclusions with perinuclear localization, whereas M1 harbored less clearly bordered inclusions of cloudy appearance (Fig 4B). Reinfection of fresh HEp-2 cells confirmed viable *Chlamydia* in lysates from M2, but not from M1 macrophages (Fig 4C). Along this line, 72±5% of M2 *vs*. 48±7% of M1 stained positive for chlamydial LPS in immunofluorescence, indicating a higher ability of M1 to control chlamydial infection (Fig 4D). To confirm these results, the expression of chlamydial 16S rDNA was determined by quantitative real-time PCR in M1 and M2 macrophages after 6 and 24 h of infection. Macrophage polarization did not influence chlamydial uptake, since the copy number of chlamydial DNA did not differ significantly for M1 and M2 after 6 h of infection. The copy number of *C*. *pneumoniae* DNA in M2 macrophages, however, increased over time (6 *vs*. 24 h post infection), while it remained unchanged in M1. This gave rise to significantly higher levels of chlamydial DNA in infected M2 macrophages as compared to M1 after 24 h. No chlamydial DNA was detected in uninfected macrophages (Fig 4E).

To investigate whether chlamydial infection would induce macrophage polarization, we infected M0 macrophages (obtained by cultivation of monocytes for 9 days in the absence of stimuli) with *C*. *pneumoniae* for 24 h. The observed cytokine secretion pattern pointed to an initial pro-inflammatory response with high release of TNF-α, followed by a trend towards M2 polarization with increasing IL-10 secretion (S1 Fig).

### Infection of M2 macrophages with *C*. *pneumoniae* enhances cytokine release

Infection of M2 macrophages with *C*. *pneumoniae* resulted in significantly enhanced release of TNF-α, IL-6, IL12p70, IL-12p40, IL-10, CCL17, and CCL24 as compared to uninfected M2 cells. Chlamydial infection of M1, in contrast, was not associated with increased cytokine secretion, most likely due to the high baseline cytokine secretion induced by LPS used for M1 differentiation.

**Fig 5:**
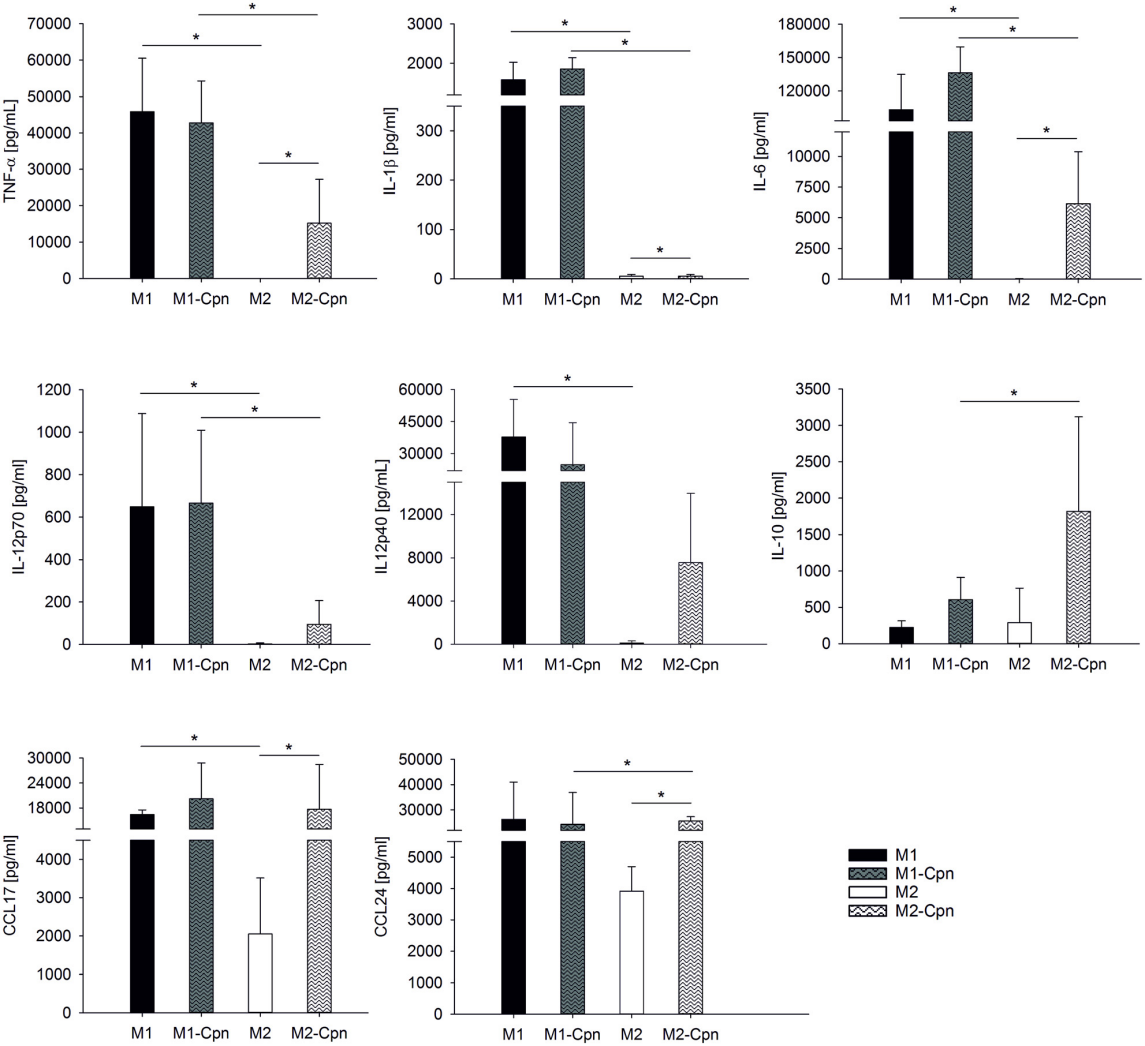
Cytokine release of M1 and M2 macrophages infected with *C*. *pneumoniae*. Monocytes were cultured and polarized as shown in Scheme 1B and fully polarized M1 and M2 macrophages (4x10^5^/mL each) were infected with *C*. *pneumoniae* (4x10^4^ IFU) for 24 h. Cytokine concentrations are expressed as mean ± SD for 3 independent experiments.

Infected M1 released higher amounts of cytokines than infected M2, with the exception of the anti-inflammatory mediator IL-10, which was released to a significantly higher extent by infected M2. No difference in secretion of the chemokines CCL17 and CCL24 was observed between infected M1 and M2 macrophages (Fig 5).

### *C*. *pneumoniae* proliferate in M1-like as well as in M2-like macrophages

**Fig 6:**
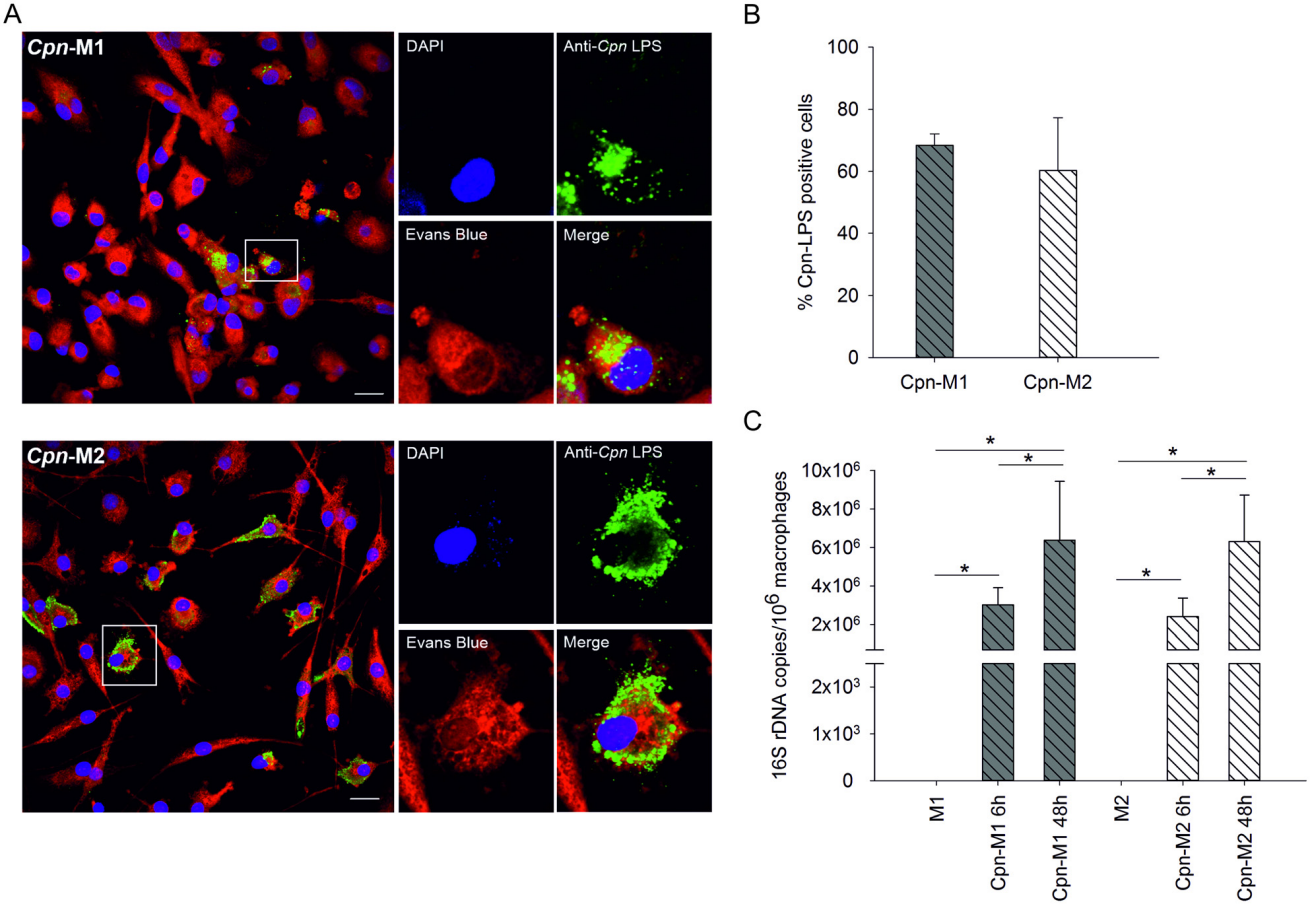
Detection of *C*. *pneumoniae* in M1-like and M2-like macrophages. M1-like and M2-like macrophages (4x10^5^/mL each) were polarized as shown in Scheme 1C and were infected with *C*. *pneumoniae* (4x10^4^ IFU) for 24 h. *C*. *pneumoniae* (green) was detected in M1-like (panel A, top) and M2-like macrophages (panel A, bottom) at 48 h post infection using immunofluorescence (scale bar = 20 μm). Cells were counterstained with Evans Blue (red), and DNA was visualized with DAPI (blue). The percentage of infected M1-like and M2-like macrophages at 48 h post infection was calculated by counting a minimum of 100 cells per slide (panel B). *C*. *pneumoniae* 16S rDNA was quantified by real-time PCR in infected M1-like and M2-like macrophages and in uninfected cells at 6 and 48 hours post infection. Data are expressed as mean ± SD for 3 independent experiments.

To assess whether the capability of fully polarized M1 macrophages to restrict chlamydial replication was also present at the level of M1-like macrophages, M1-like and M2-like macrophages were infected with *C*. *pneumoniae*, and chlamydial load was analyzed by immunofluorescence and real-time PCR (Fig 6). About 60±17% of all cells stained positive for chlamydial LPS, with no difference between M1-like and M2-like macrophages (Fig 6B). Comparable amounts of chlamydial LPS were present in M1-like and M2-like cells after 48 h, but chlamydial LPS seemed to be spread in the cytoplasm in M2-like macrophages, while chlamydial inclusions appeared as dense aggregates in the cytoplasm of M1-like macrophages (Fig 6A).

Real-time PCR showed no differences in the copy numbers of intracellular *C*. *pneumoniae* between M1-like and M2-like macrophages after 6 h of infection, providing further evidence that chlamydial uptake did not differ for M1-like and M2-like macrophages. Chlamydial load increased over time in both M1-like and M2-like cells, indicating replication of *C*. *pneumoniae* in both M1-like and M2-like macrophages, while no chlamydial DNA was detected in uninfected macrophages (Fig 6C).

To further examine the influence of LPS used during M1 polarization on cytokine secretion (see above), we used M1-like macrophages cultivated in the absence of LPS and found that they were fully reactive to *C*. *pneumoniae* infection. In analogy with fully polarized M1, infected M1-like macrophages showed higher cytokine release than infected M2-like macrophages, again with the exception of the anti-inflammatory mediator IL-10 which was released to a significantly higher extent by infected M2-like cells (S2 Fig).

## Discussion

Macrophages represent a diverse population of phagocytic cells residing in tissues throughout the body, providing an environment for survival and replication of a number of intracellular bacteria. Several studies have demonstrated persistence of *C*. *pneumoniae* in primary human monocytes as well as replication in monocyte-derived macrophages [9, 11, 27, 28]. Findings from an *in vivo* mouse model using *C*. *pneumoniae* indicate an impact of macrophage polarization on the course of chronic lung inflammation, suggesting that M1 macrophages result in enhanced inflammation, tissue injury, and fibrosis [29]. Moreover, it has recently been shown in a murine model *in vitro* that the intracellular survival and persistence of *C*. *muridarum* in mouse macrophages is determined by their phenotypic plasticity [23]. The interdependence of *chlamydial* infection and macrophage polarization in the human system, however, is still unknown.

To explore the ability of *C*. *pneumoniae* to survive in polarized human macrophages and to assess the influence of macrophage polarization on the control of chlamydial infection, we pre-differentiated human monocytes with GM-CSF and M-CSF for 7 days, followed by treatment of the resulting M1-like and M2-like macrophages with IFN-γ/LPS or IL-4 for 48 h to yield M1 and M2 macrophages. It is well established that colony-stimulating factors induce functional heterogeneity in monocyte-derived macrophages [30–32], and pre-differentiation with GM-CSF and M-CSF has been demonstrated to enhance the final M1/M2 activation status [33]. Since pro-inflammatory M1 and anti-inflammatory M2 macrophages represent two poles of a continuum of overlapping cellular activities [22, 34, 35], we used a combination of morphological parameters, surface marker expression, and cytokine secretion [36] to define the polarization outcomes and to characterize the macrophage subpopulations. Fully polarized M1 and M2 macrophages were clearly discriminated by their morphology, as shown by microscopy as well as by flow cytometric analysis of their forward and side scatter characteristics. M1 and M2 polarization resulted in distinct surface marker profiles with high expression of the co-stimulatory molecule CD86 on M1 and high levels of CD11b, CD163, as well as the mannose-binding lectin receptors CD206 and CD209 on M2 macrophages. Contrasting published data for human macrophages [32], where CD206 did not discriminate between M1 and M2 polarization, we observed comparable CD206 expression on pre-polarized M1-like and M2-like macrophages, but strong CD206 up-regulation during full M2 polarization. While differences in the polarization protocols, in particular the length of the polarization phase, may account for these divergences, they also illustrate the dynamics of macrophage polarization, which is crucially influenced by the microenvironment and may result in mixed phenotypes with co-existence of M1 and M2 signatures [37]. Complementing morphological characterization and surface marker expression, we determined cytokine release to define polarization outcomes. Cytokine secretion was very low or completely lacking in M1-like and M2-like macrophages, while M1 polarization was associated with release of high amounts of TNF-α, IL-1β, IL-6, IL12p70, and IL-12p40. Remarkably, the anti-inflammatory mediator IL-10 was upregulated in M1, but not in M2 macrophages; yet this confirms previous findings in murine and human models [23, 38].

M1 and M2 polarization did not affect uptake of *C*. *pneumoniae*, as revealed by the presence of comparable copy numbers of *C*. *pneumoniae* DNA in M1 and M2 macrophages at 6 h post infection, but an increase in copy numbers over time indicating proliferation of *C*. *pneumoniae* was detected for M2 macrophages only, while copy numbers in M1 macrophages remained stable. Accordingly, a significantly higher percentage of M2 stained positive for chlamydial LPS in immunofluorescence at 24 h post infection. The morphology of chlamydial inclusions was clearly different for M1 and M2, with typical large inclusions nestled in the perinuclear region in M2 cells and diffuse, cloudy, non-perinuclear inclusions in M1 cells. Large chlamydial inclusions have previously been described in M2 (23), and the perinuclear localization may provide factors essential for chlamydial development [39]. Reinfection of HEp-2 cells with lysates from M1 and M2 confirmed the presence of viable *C*. *pneumoniae* in M2, but not in M1 macrophages.

In line with data from a murine model [23], these findings provide strong evidence for the enhanced ability of M1 to control intracellular bacteria also in the human system. Interferon-γ-mediated microbicidal activity with high levels of pro-inflammatory cytokines, production of reactive oxygen species, iNOS-dependent reactive nitrogen intermediates, as well as indoleamine 2,3-dioxygenase leading to tryptophan depletion [40–42] may explain the restriction of chlamydial replication in M1. Pre-polarized M1-like and M2-like macrophages, in contrast, did not differ with respect to their ability to control chlamydial infection, since chlamydial DNA increased over time in both M1-like and M2-like cells, and equal percentages of M1-like and M2-like macrophages stained positive for chlamydial LPS.

While our data support the notion that M2 polarization favors survival and replication of *C*. *pneumoniae*, we did not find definite evidence that *C*. *pneumoniae* would interfere with M1 polarization or would induce an M2 phenotype in macrophages, since infection of M0 macrophages generated by cultivation of monocytes in the absence of stimuli for 9 days elicited an initial pro-inflammatory response with high release of TNF-α and IL-12, followed by high IL-10 release at later time points.

In conclusion, our findings support the relevance of macrophage polarization in the control of chlamydial infection and are the first demonstration in the human system that M2 macrophages are the preferred niche for survival and replication of the intracellular pathogen *C*. *pneumoniae*. The fact that M1 macrophages failed to completely eliminate chlamydial infection underscores the ability of *C*. *pneumoniae* to evade cellular defense and to persist in host macrophages over time.

## Supporting Information

S1 Fig Infection of M0 macrophages with *C*. *pneumoniae*. Monocytes (1x10^6^ cells/mL) were cultured in RPMI-1640/10%FBS for 7 days. After harvesting, 4x10^5^/mL macrophages were resuspended in MSF medium/2% AB serum without antibiotics and cultured 48 hours. M0 macrophages were infected with *C*. *pneumoniae* (4x10^4^ IFU) for 24 hours. Cytokine secretion of infected M0 macrophages after 6 and 24 hours was quantified using the Bio-Plex 200 system (Fig A). Morphology was assessed using light microscopy; scale bar: 200 μm (Fig B). *C*. *pneumoniae* 16S rDNA copies in infected M0 macrophages and uninfected cells were quantified at 6 and 24 hours post infection by real-time PCR (Fig C). Concentrations are expressed as mean ± SD for 3 independent experiments.*P ≤ 0.05
(PDF)

S2 Fig Cytokine release of M1-like and M2-like macrophages infected with *C*. *pneumoniae*. Monocytes were cultured and polarized as shown in Scheme 1C. M1-like and M2-like macrophages (4x10^5^/mL each) were infected with *C*. *pneumoniae* (4x10^4^ IFU) for 48 h. Cytokine concentrations are expressed as mean ± SD for 3 independent experiments.
(PDF)

## References

[1] Price JV, Vance RE. The Macrophage Paradox. Immunity. 2014;41(5):685–93. 10.1016/j.immuni.2014.10.015 . 25517611

[2] Thi EP, Lambertz U, Reiner NE. Sleeping with the enemy: how intracellular pathogens cope with a macrophage lifestyle. PLoS pathogens. 2012;8(3):e1002551 10.1371/journal.ppat.1002551 22457616 PMC3310772

[3] Ray K, Marteyn B, Sansonetti PJ, Tang CM. Life on the inside: the intracellular lifestyle of cytosolic bacteria. Nature reviews Microbiology. 2009;7(5):333–40. 10.1038/nrmicro2112 . 19369949

[4] Kern JM, Maass V, Maass M. Molecular pathogenesis of chronic Chlamydia pneumoniae infection: a brief overview. Clinical microbiology and infection: the official publication of the European Society of Clinical Microbiology and Infectious Diseases. 2009;15(1):36–41. 10.1111/j.1469-0691.2008.02631.x . 19220338

[5] Saikku P. Seroepidemiology in Chlamydia pneumoniae—atherosclerosis association. European heart journal. 2002;23(4):263–4. 10.1053/euhj.2001.2913 . 11812058

[6] von HL. Role of persistent infection in the control and severity of asthma: focus on Chlamydia pneumoniae. The European respiratory journal. 2002;19(3):546–56. . 11936537 10.1183/09031936.02.00254402

[7] Balin BJ, Little CS, Hammond CJ, Appelt DM, Whittum-Hudson JA, Gerard HC, et al Chlamydophila pneumoniae and the etiology of late-onset Alzheimer's disease. Journal of Alzheimer's disease: JAD. 2008;13(4):371–80. . 18487846 10.3233/jad-2008-13403

[8] Wyrick PB. Chlamydia trachomatis persistence in vitro: an overview. J Infect Dis. 2010;201 Suppl 2:S88–95. Epub 2010/05/28. 10.1086/652394 20470046 PMC2878585

[9] Buchacher T, Wiesinger-Mayr H, Vierlinger K, Ruger BM, Stanek G, Fischer MB, et al Human blood monocytes support persistence, but not replication of the intracellular pathogen C. pneumoniae. BMC immunology. 2014;15(1):60 10.1186/s12865-014-0060-1 25488836 PMC4268907

[10] Wolf K, Fischer E, Hackstadt T. Degradation of Chlamydia pneumoniae by peripheral blood monocytic cells. Infection and immunity. 2005;73(8):4560–70. 10.1128/IAI.73.8.4560-4570.2005 16040967 PMC1201216

[11] Airenne S, Surcel HM, Alakarppa H, Laitinen K, Paavonen J, Saikku P, et al Chlamydia pneumoniae infection in human monocytes. Infection and immunity. 1999;67(3):1445–9. 10024593 10.1128/iai.67.3.1445-1449.1999 PMC96479

[12] Biswas SK, Mantovani A. Macrophage plasticity and interaction with lymphocyte subsets: cancer as a paradigm. Nature immunology. 2010;11(10):889–96. 10.1038/ni.1937 . 20856220

[13] Murray PJ, Wynn TA. Protective and pathogenic functions of macrophage subsets. Nature reviews Immunology. 2011;11(11):723–37. 10.1038/nri3073 21997792 PMC3422549

[14] Gordon S. Alternative activation of macrophages. Nature reviews Immunology. 2003;3(1):23–35. 10.1038/nri978 . 12511873

[15] Biswas SK, Mantovani A. Orchestration of metabolism by macrophages. Cell metabolism. 2012;15(4):432–7. 10.1016/j.cmet.2011.11.013 . 22482726

[16] Ghesquiere B, Wong BW, Kuchnio A, Carmeliet P. Metabolism of stromal and immune cells in health and disease. Nature. 2014;511(7508):167–76. 10.1038/nature13312 . 25008522

[17] Abdullah Z, Geiger S, Nino-Castro A, Bottcher JP, Muraliv E, Gaidt M, et al Lack of PPARgamma in myeloid cells confers resistance to Listeria monocytogenes infection. PloS one. 2012;7(5):e37349 10.1371/journal.pone.0037349 22629382 PMC3357414

[18] Ketavarapu JM, Rodriguez AR, Yu JJ, Cong Y, Murthy AK, Forsthuber TG, et al Mast cells inhibit intramacrophage Francisella tularensis replication via contact and secreted products including IL-4. Proceedings of the National Academy of Sciences of the United States of America. 2008;105(27):9313–8. 10.1073/pnas.0707636105 18591675 PMC2453703

[19] Rajaram MV, Brooks MN, Morris JD, Torrelles JB, Azad AK, Schlesinger LS. Mycobacterium tuberculosis activates human macrophage peroxisome proliferator-activated receptor gamma linking mannose receptor recognition to regulation of immune responses. Journal of immunology. 2010;185(2):929–42. 10.4049/jimmunol.1000866 20554962 PMC3014549

[20] Rodriguez AR, Yu JJ, Murthy AK, Guentzel MN, Klose KE, Forsthuber TG, et al Mast cell/IL-4 control of Francisella tularensis replication and host cell death is associated with increased ATP production and phagosomal acidification. Mucosal immunology. 2011;4(2):217–26. 10.1038/mi.2010.59 20861832 PMC3040285

[21] Mahajan S, Dkhar HK, Chandra V, Dave S, Nanduri R, Janmeja AK, et al Mycobacterium tuberculosis modulates macrophage lipid-sensing nuclear receptors PPARgamma and TR4 for survival. Journal of immunology. 2012;188(11):5593–603. 10.4049/jimmunol.1103038 . 22544925

[22] Benoit M, Desnues B, Mege JL. Macrophage polarization in bacterial infections. Journal of immunology. 2008;181(6):3733–9. . 18768823 10.4049/jimmunol.181.6.3733

[23] Gracey E, Lin A, Akram A, Chiu B, Inman RD. Intracellular survival and persistence of Chlamydia muridarum is determined by macrophage polarization. PloS one. 2013;8(8):e69421 10.1371/journal.pone.0069421 23967058 PMC3743904

[24] Datta B, Njau F, Thalmann J, Haller H, Wagner AD. Differential infection outcome of Chlamydia trachomatis in human blood monocytes and monocyte-derived dendritic cells. BMC microbiology. 2014;14:209 10.1186/s12866-014-0209-3 25123797 PMC4236547

[25] Sommer K, Njau F, Wittkop U, Thalmann J, Bartling G, Wagner A, et al Identification of high- and low-virulent strains of Chlamydia pneumoniae by their characterization in a mouse pneumonia model. FEMS immunology and medical microbiology. 2009;55(2):206–14. 10.1111/j.1574-695X.2008.00503.x . 19076226

[26] Goldschmidt P, Rostane H, Sow M, Goepogui A, Batellier L, Chaumeil C. Detection by broad-range real-time PCR assay of Chlamydia species infecting human and animals. The British journal of ophthalmology. 2006;90(11):1425–9. 10.1136/bjo.2006.096420 16899531 PMC1857507

[27] Rupp J, Pfleiderer L, Jugert C, Moeller S, Klinger M, Dalhoff K, et al Chlamydia pneumoniae hides inside apoptotic neutrophils to silently infect and propagate in macrophages. PloS one. 2009;4(6):e6020 10.1371/journal.pone.0006020 19547701 PMC2695784

[28] Marangoni A, Bergamini C, Fato R, Cavallini C, Donati M, Nardini P, et al Infection of human monocytes by Chlamydia pneumoniae and Chlamydia trachomatis: an in vitro comparative study. BMC research notes. 2014;7:230 10.1186/1756-0500-7-230 24721461 PMC3984436

[29] Jupelli M, Shimada K, Chiba N, Slepenkin A, Alsabeh R, Jones HD, et al Chlamydia pneumoniae infection in mice induces chronic lung inflammation, iBALT formation, and fibrosis. PLoS One. 2013;8(10):e77447 10.1371/journal.pone.0077447 24204830 PMC3808399

[30] Lenzo JC, Turner AL, Cook AD, Vlahos R, Anderson GP, Reynolds EC, et al Control of macrophage lineage populations by CSF-1 receptor and GM-CSF in homeostasis and inflammation. Immunology and cell biology. 2012;90(4):429–40. 10.1038/icb.2011.58 . 21727904

[31] Hamilton JA. Colony-stimulating factors in inflammation and autoimmunity. Nature reviews Immunology. 2008;8(7):533–44. 10.1038/nri2356 . 18551128

[32] Jaguin M, Houlbert N, Fardel O, Lecureur V. Polarization profiles of human M-CSF-generated macrophages and comparison of M1-markers in classically activated macrophages from GM-CSF and M-CSF origin. Cellular immunology. 2013;281(1):51–61. 10.1016/j.cellimm.2013.01.010 . 23454681

[33] Mia S, Warnecke A, Zhang XM, Malmstrom V, Harris RA. An optimized protocol for human M2 macrophages using M-CSF and IL-4/IL-10/TGF-beta yields a dominant immunosuppressive phenotype. Scandinavian journal of immunology. 2014;79(5):305–14. 10.1111/sji.12162 24521472 PMC4282403

[34] Mosser DM, Edwards JP. Exploring the full spectrum of macrophage activation. Nature reviews Immunology. 2008;8(12):958–69. 10.1038/nri2448 19029990 PMC2724991

[35] Geissmann F, Gordon S, Hume DA, Mowat AM, Randolph GJ. Unravelling mononuclear phagocyte heterogeneity. Nature reviews Immunology. 2010;10(6):453–60. 10.1038/nri2784 20467425 PMC3032581

[36] Murray PJ, Allen JE, Biswas SK, Fisher EA, Gilroy DW, Goerdt S, et al Macrophage activation and polarization: nomenclature and experimental guidelines. Immunity. 2014;41(1):14–20. 10.1016/j.immuni.2014.06.008 25035950 PMC4123412

[37] Martinez FO, Gordon S. The M1 and M2 paradigm of macrophage activation: time for reassessment. F1000prime reports. 2014;6:13 10.12703/P6-13 24669294 PMC3944738

[38] Lopez-Castejon G, Baroja-Mazo A, Pelegrin P. Novel macrophage polarization model: from gene expression to identification of new anti-inflammatory molecules. Cellular and molecular life sciences: CMLS. 2011;68(18):3095–107. 10.1007/s00018-010-0609-y . 21188461 PMC11114961

[39] Cocchiaro JL, Valdivia RH. New insights into Chlamydia intracellular survival mechanisms. Cellular microbiology. 2009;11(11):1571–8. 10.1111/j.1462-5822.2009.01364.x 19673891 PMC2787098

[40] Rottenberg ME, Gigliotti Rothfuchs A, Gigliotti D, Ceausu M, Une C, Levitsky V, et al Regulation and role of IFN-gamma in the innate resistance to infection with Chlamydia pneumoniae. Journal of immunology. 2000;164(9):4812–8. . 10779789 10.4049/jimmunol.164.9.4812

[41] Rottenberg ME, Gigliotti-Rothfuchs A, Wigzell H. The role of IFN-gamma in the outcome of chlamydial infection. Current opinion in immunology. 2002;14(4):444–51. . 12088678 10.1016/s0952-7915(02)00361-8

[42] Muraille E, Leo O, Moser M. TH1/TH2 paradigm extended: macrophage polarization as an unappreciated pathogen-driven escape mechanism? Frontiers in immunology. 2014;5:603 10.3389/fimmu.2014.00603 25505468 PMC4244692

