# Supplementary figures and images for: M2 Polarization of Human Macrophages Favors Survival of the Intracellular Pathogen Chlamydia pneumoniae
Source: PLoS One. 2015 Nov 25;10(11):e0143593. doi: 10.1371/journal.pone.0143593 (PMC4659546; doi:10.1371/journal.pone.0143593)

Supporting Information

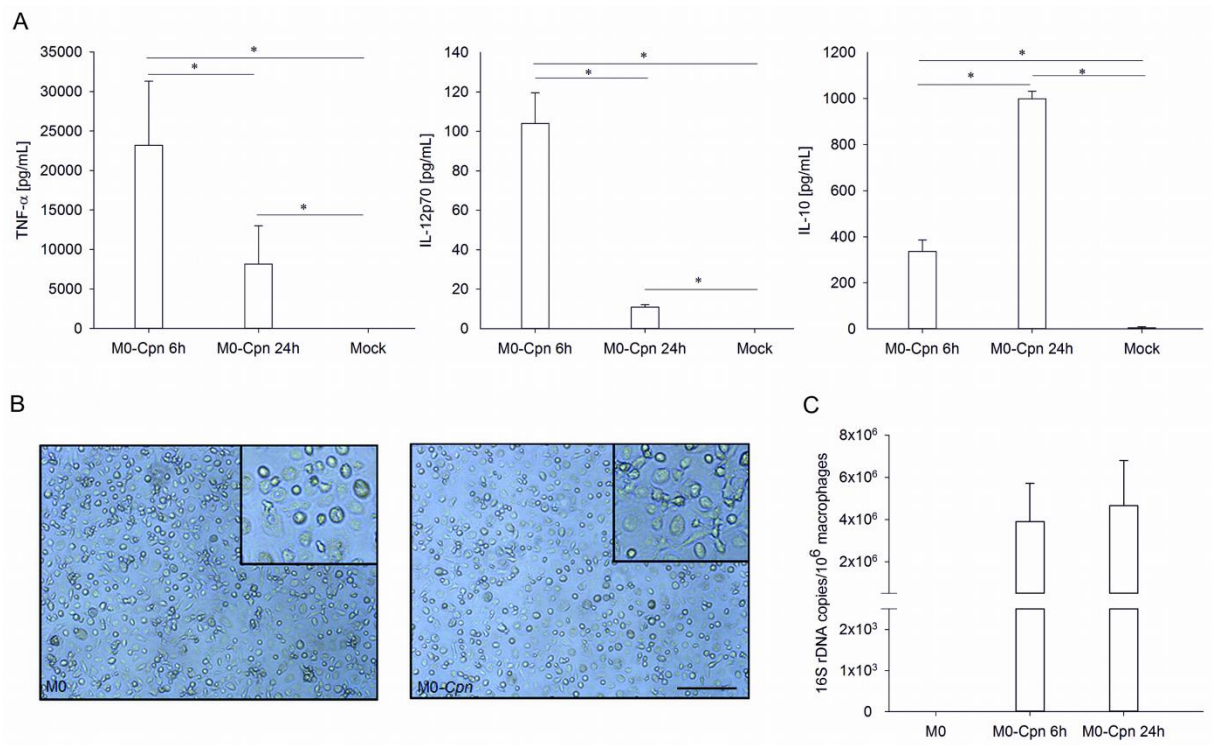

Figure S1

Supplement: S1 Fig — Monocytes (1x10^6^ cells/mL) were cultured in RPMI-1640/10%FBS for 7 days. After harvesting, 4x10^5^/mL macrophages were resuspended in MSF medium/2% AB serum without antibiotics and cultured 48 hours. M0 macrophages were infected with C. pneumoniae (4x10^4^ IFU) for 24 hours. Cytokine secretion of infected M0 macrophages after 6 and 24 hours was quantified using the Bio-Plex 200 system (Fig A). Morphology was assessed using light microscopy; scale bar: 200 μm (Fig B). C. pneumoniae 16S rDNA copies in infected M0 macrophages and uninfected cells were quantified at 6 and 24 hours post infection by real-time PCR (Fig C). Concentrations are expressed as mean ± SD for 3 independent experiments.*P ≤ 0.05 (PDF) [file pone.0143593.s001.pdf]

Supporting Information

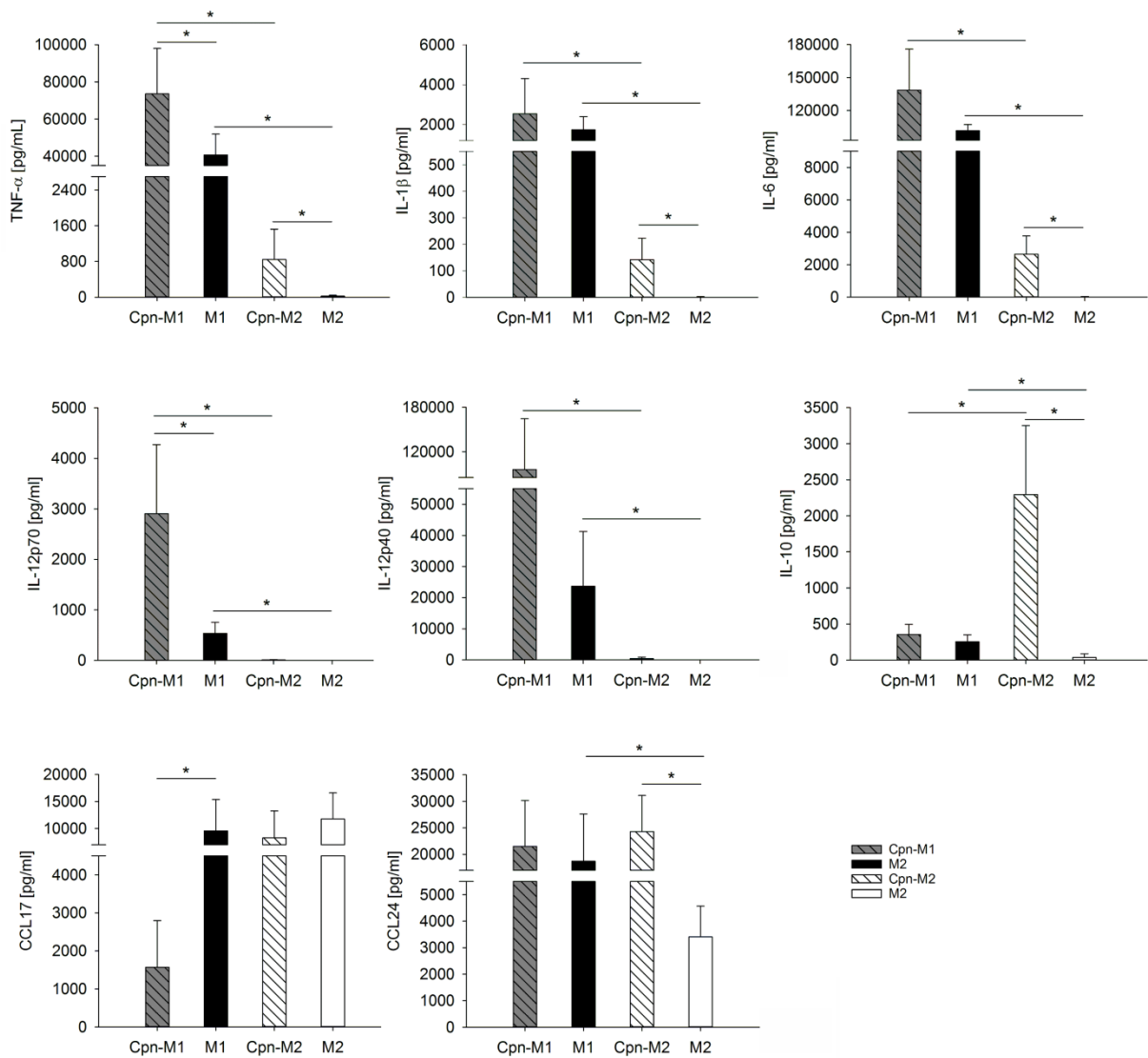

Figure S2

Supplement: S2 Fig — Monocytes were cultured and polarized as shown in Scheme 1C. M1-like and M2-like macrophages (4x10^5^/mL each) were infected with C. pneumoniae (4x10^4^ IFU) for 48 h. Cytokine concentrations are expressed as mean ± SD for 3 independent experiments. (PDF) [file pone.0143593.s002.pdf]
